# Effect of statin use on clinical outcomes in ischemic stroke patients with atrial fibrillation

**DOI:** 10.1097/MD.0000000000005918

**Published:** 2017-02-03

**Authors:** Yi-Ling Wu, Jeffrey L. Saver, Pei-Chun Chen, Jiann-Der Lee, Hui-Hsuan Wang, Neal M. Rao, Meng Lee, Bruce Ovbiagele

**Affiliations:** a Department of Neurology, Chang Gung University College of Medicine, Chang Gung Memorial Hospital, Chiayi Branch; b Department of Public Health, China Medical University, Taichung, Taiwan; c Stroke Center, Geffen School of Medicine, University of California, Los Angeles, CA; d Department of Healthcare Management, Chang Gung University, Taoyuan, Taiwan; e Department of Neurosciences, Medical University of South Carolina, Charleston, South Carolina, SC.

**Keywords:** atrial fibrillation, ischemic stroke, outcome, statin

## Abstract

It remains unclear whether statin therapy should be applied to ischemic stroke patients with atrial fibrillation. The objective of this study was to clarify whether statin therapy can influence the prognosis in recent ischemic stroke patients with atrial fibrillation.

We identified ischemic stroke patients with atrial fibrillation between 2001 and 2011 from Taiwan National Health Insurance Database. Patients not treated with statins during the first 90 days after the index stroke were matched to patients treated with statins in the first 90 days in a 2:1 ratio on the basis of age, sex, hypertension, diabetes mellitus, ischemic heart disease, heart failure, estimated National Institutes of Health Stroke Scale, use of anticoagulant, and year of their entry into the cohort. The primary outcome was the first event of recurrent stroke, and the secondary outcome was in-hospital death.

A total of 1546 atrial fibrillation patients with statin therapy in the first 90 days poststroke and 3092 matched atrial fibrillation nonstatin controls were enrolled for this analysis. During the median 2.4-year follow-up, the risk of recurrent stroke was not different between subjects receiving versus not receiving statin therapy (hazard ratios = 1.01, 95% confidence interval 0.88 to 1.15). However, patients with atrial fibrillation receiving statin therapy had a reduced risk for death during any hospitalization throughout the long-term follow-up period (hazard ratios = 0.74, 95% confidence interval 0.61 to 0.89).

Among ischemic stroke patients with atrial fibrillation, statin therapy initiated during the acute to subacute poststroke stage did not alter the rate of stroke recurrence but was associated with a decreased rate of in-hospital death.

## 1 Introduction

Ischemic stroke is a disease with substantial etiologic heterogeneity, including large artery atherothrombotic disease, small artery atherothrombotic disease, cardioembolism, and other subtypes.^[1]^ HMG-CoA reductase inhibitors, or “statins,” are established therapeutic agents for the prevention of atherosclerotic-based large or small vessel vascular events.^[2]^ Indeed, in a randomized trial assessing the impact of statin use on recurrent vascular events of atherosclerotic origin, the Stroke Prevention by Aggressive Reduction in Cholesterol Levels (SPARCL) study, high dose atorvastatin significantly reduced the risk of recurrent stroke as well as recurrent major vascular events. However, in order to target recent stroke patients with atherosclerotic disease, SPARCL excluded patients with atrial fibrillation.^[3]^ Based on published data, it remains unclear as to whether statin therapy should be applied to the substantial numbers of ischemic stroke patients who have atrial fibrillation as an identified potential mechanism of their event, including patients with and without hypercholesterolemia and patients with and without evidence of tandem large or small vessel atherosclerotic disease. As far as we are aware, there are no ongoing clinical trials designed to address this issue.

Clarifying whether statins are beneficial in atrial fibrillation patients with ischemic stroke would assist with a common management dilemma. Among ischemic stroke patients in the Taiwan Stroke Registry, 16.5% had atrial fibrillation, and 49.4% had dyslipidemia, with 8% of these patients having both atrial fibrillation and hypercholesteremia.^[4]^ Beside their effect of lowering cholesterol to mitigate symptomatic atherosclerosis, statins have pleiotropic antithrombotic, anti-inflammatory, and additional actions that also may be beneficial in lowering vascular risk in patients with atrial fibrillation. For instance, atrial fibrillation upregulates CD40 expression and platelet adhesion to the endocardium, and simvastatin is effective in modulating this expression, potentially reducing the risk of intra-atrial thrombus formation.^[5]^ Also, a randomized controlled trial enrolling 34 elderly patients with atrial fibrillation showed that intensive cholesterol lowering with atorvastatin significantly reduced inflammation and was accompanied by reduced thrombin generation.^[6]^

To investigate whether statin therapy can influence the prognosis, such as recurrent stroke and mortality, in recent ischemic stroke patients with atrial fibrillation, we conducted a retrospective cohort study in Taiwan using the National Health Insurance Research Database (NHIRD).

## 2 Methods

### 2.1 Study design and dataset

We retrospectively assessed data collected between 2001 through 2012, from the Taiwan NHIRD.

Taiwan implemented a single-payer, compulsory National Health Insurance program in 1995, which includes reimbursement of outpatient visits, hospital admissions, and prescriptions for 99% of the Taiwanese population. All contracted institutions must file claims according to standard formats, which later are archived in the NHIRD.

### 2.2 Study population

We identified all hospitalized patients (≥18 years) who were admitted with a primary diagnosis of ischemic stroke (International Classification of Diseases, Ninth Revision (ICD-9) codes 433, 434, 436) for the first time between 2001 and 2011. The first ischemic stroke during the study period was defined as the index stroke. Only patients who had atrial fibrillation (ICD-9 code 427.31) documented prior to the index stroke or in a list of hospitalization diagnoses during the index stroke admission were included in our analysis. In order to show the effect of statin therapy, patients were excluded if they had a recurrent stroke ≤90 days after the index stroke or follow-up ≤90 days. We also excluded patients who undergo hemodialysis because it is still unclear whether statin is benefit for these patients.^[7]^ This is a nationwide study that included all available and eligible patients.

Patients receiving at least 30 days of statin therapy within 90 days after the index stroke were categorized in the statin group, whereas patients not receiving any statin therapy within 90 days were categorized in a comparison group. Patients receiving some statin therapy, but less than 30 days, within 90 days after index stroke were excluded. During the study period, the national reimbursement policy for statin prescriptions supported statin prescriptions for patients with total cholesterol ≧200 mg/dL or low-density lipoprotein cholesterol ≧130 mg/dL. Comorbidities were identified using ICD-9 codes based on coding of concomitant diagnoses during the index stroke admission and on coding for outpatient visit before the index stroke. Heart failure was diagnosed solely from the hospitalization diagnoses since coding for this diagnosis is less reliable in the outpatient setting in Taiwan. The Stroke Severity Index, which was developed specifically to evaluate the severity of strokes in Taiwan NHIRD, was used to generate an estimated National Institutes of Health Stroke Scale (e-NIHSS) score for the index stroke.^[8]^ The Pearson correlation coefficient between the e-NIHSS and the NIHSS real scores was 0.742 (95% CI: 0.736, 0.747).^[8]^

Patients not treated with statins in the first 90 days after the index stroke were matched to patients treated with statins in the first 90 days in a 2:1 ratio on the basis of age, sex, hypertension, diabetes mellitus, ischemic heart disease, heart failure, e-NIHSS, use of anticoagulant (warfarin, dabigatran, and rivaroxaban) in the first 90 days after index stroke, and year of their entry into the cohort. The study protocol was approved by the Institutional Review Boards of Chang Gung Memorial Hospital.

### 2.3 Main outcome measures

The primary outcome of this study was the first event of recurrent stroke (combined endpoint of ischemic and hemorrhagic stroke), validated by the use of brain computed tomography or magnetic resonance imaging. The requirement for imaging served to exclude stroke patients who were hospitalized solely for rehabilitation during chronic stage. The leading secondary outcome was in-hospital death, defined as any death during a hospitalization during the follow-up period. Deaths occurring out of hospital were not ascertained. Additional outcomes were hemorrhagic stroke (ICD-9 codes 430–432), ischemic stroke (ICD-9 codes 433–434, 436), fatal stroke, myocardial infarction (ICD-9 code 410), and major adverse cardiovascular events (comprised of any ischemic or hemorrhagic stroke, and myocardial infarction). Causes of death were defined according to the primary diagnosis of the hospitalization during which the death occurred. Fatal stroke was defined as in-hospital death during the first recurrent ischemic or hemorrhagic stroke. Cardiovascular death was defined as in-hospital death caused by stroke (ICD-9 codes 430–438), ischemic heart disease (ICD-9 codes 410–414), cardiac arrhythmia (ICD-9 codes 425–427), or heart failure (ICD-9 code 428). Noncardiovascular death was defined as in-hospital death with primary diagnosis other than cardiovascular death. We also analyzed the subgroup of patients not having diabetes mellitus at baseline to see whether statin therapy was associated with increased incident diabetes mellitus.

The follow-up period for outcome events started from the date of index stroke to the date of last medical claim, stroke recurrence, death during any hospitalization, or the end of 2012, whichever came first.

### 2.4 Statistical analysis

The association between statin therapy and recurrent stroke in stroke patients with atrial fibrillation was analyzed using the Cox proportional hazards model and presented as hazard ratios (HR) with 95% confidence intervals (95% CI), and so did subgroups and interactions. All statistics analyses were performed with SAS statistical software, version 9.2 (SAS Institute Inc., Cary, NC). A 2-sided *P* value < 0.05 was considered to be statistically significant.

## 3 Results

A total of 1546 patients with statin therapy in the first 90 days poststroke and 3092 matched controls were enrolled for this analysis. The mean age was 75.6 ± 7.4 years, and approximately 49% of them were male (Table 1). Overall, the index ischemic stroke hospitalization occurred from 2001 to 2006 in 31% of patients and from 2007 to 2011 in 69%. (There were twice as many patients from the later 5-year period than from the earlier 4-year period because statin was much more widely used after the publication of the SPARCL trial in 2006).^[3]^

**Table 1:**
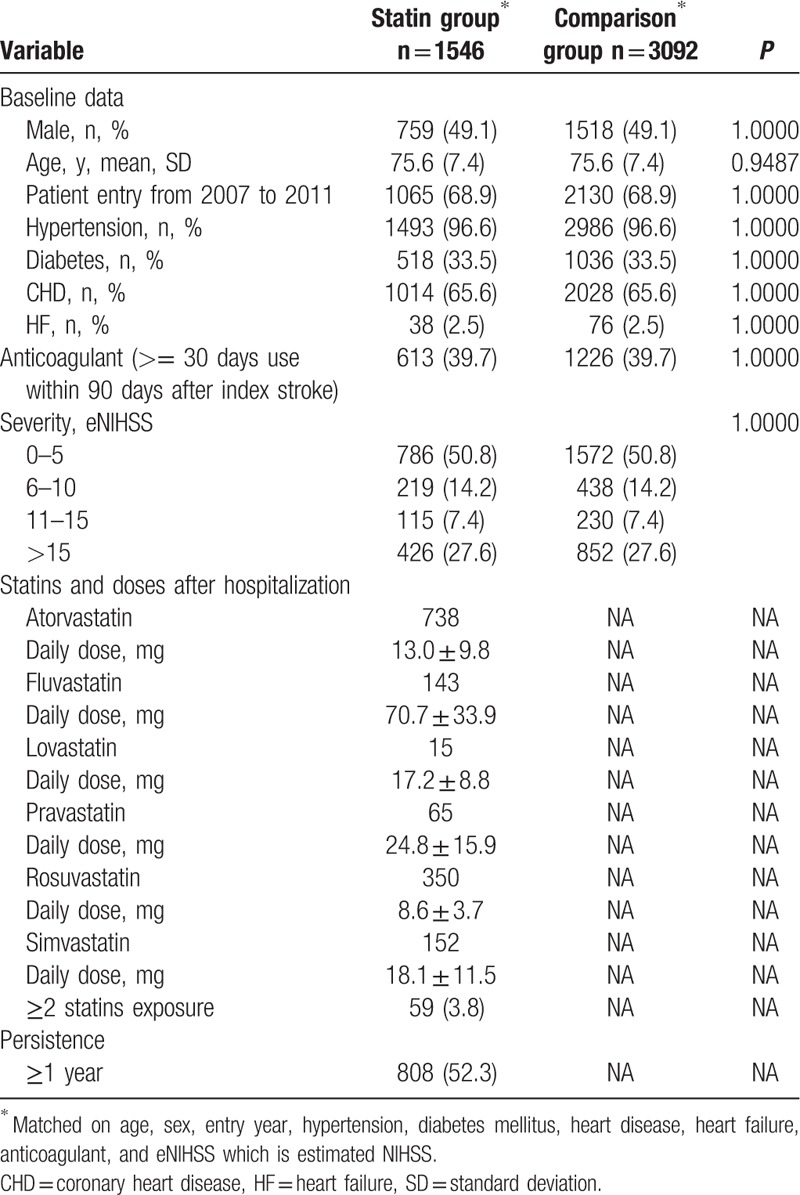
Baseline characteristic of the subjects.

About 40% of patients received anticoagulant therapy for recurrent stroke prevention. Index ischemic stroke severity was minor (e-NIHSS 0–5) in half of the subjects, moderate (e-NIHSS 6–10) in 14%, moderate to severe (e-NIHSS 11–15) in 7%, and severe (e-NIHSS >15) in 28%. The baseline characteristics were not different between the 2 groups as the result of matching. In the early statin group, 3.8% of subjects had received 2 or more statins during the first 90 poststroke days.

The follow-up period was from 0.3 year to 12 year (median 2.4 years; interquartile range 1.3–4.1 years). During this period, there were 933 events of first recurrent ischemic or hemorrhagic strokes. The results revealed no difference in stroke recurrence between early as far as statin-users and early nonusers (HR = 1.01, 95% CI 0.88–1.15, *P* = 0.92). For the secondary endpoint, early statin use was associated with lower frequency of death during any hospitalization throughout the long-term follow-up period (HR = 0.74, 95% CI 0.61–0.89, *P* < 0.01) (Table 2). This difference was due to a reduced rate of noncardiovascular death (HR = 0.70, 95% CI 0.56–0.86, *P* < 0.01) Causes of noncardiovascular death in the 2 groups are shown in Table 3. The effects of statin therapy were similar across the additional endpoints, including ischemic stroke, intracerebral hemorrhage, fatal stroke, myocardial infarction, and major adverse cardiovascular events. Among patients not having diabetes mellitus at baseline, early post-stroke statin therapy was not associated with increased incidence of diabetes mellitus (Table 2).

**Table 2:**
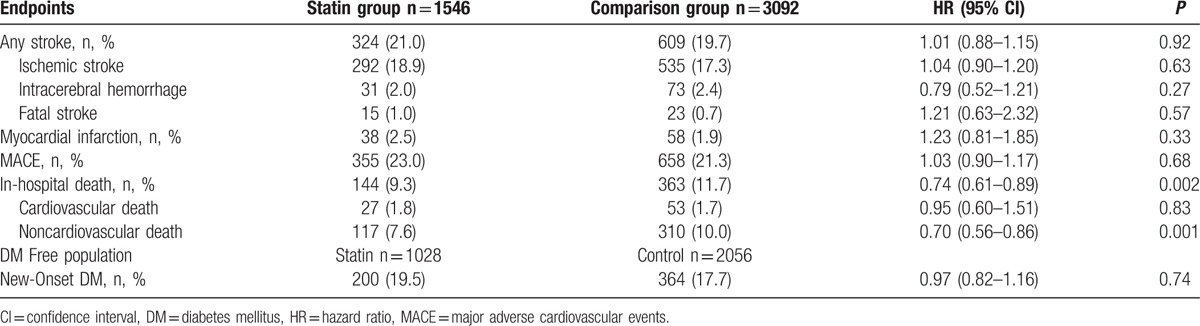
Cox proportional Hazard models for primary and secondary end points by statin group vs comparison group.

**Table 3:**
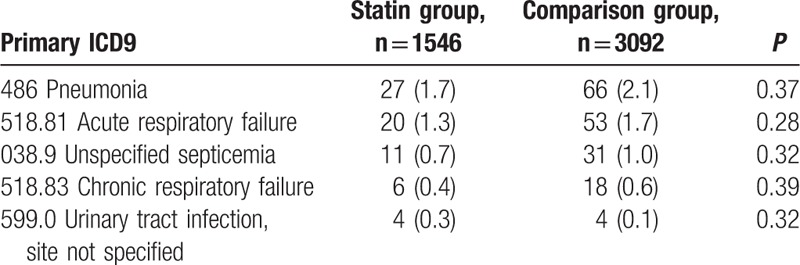
Primary ICD9 code of noncardiovascular death.

**Figure 1:**
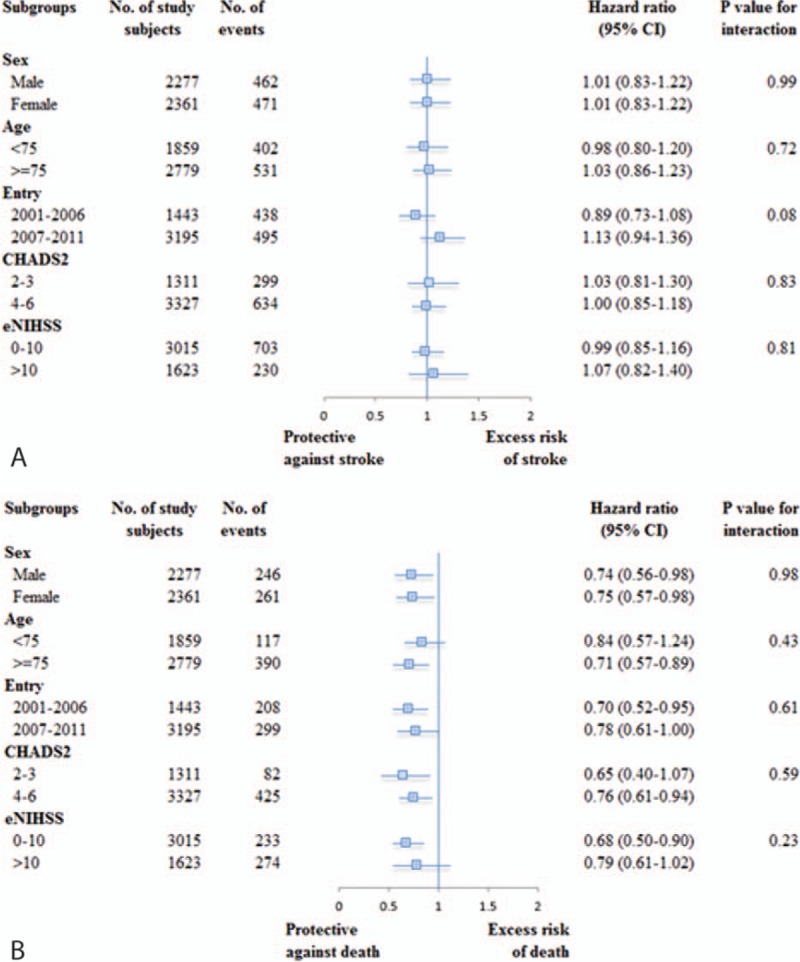
Subgroup analysis for recurrent stroke (A) and in-hospital death (B) between statin and comparison groups.

The effects of early statin therapy were consistent across subgroups of age, sex, calendar year, CHADS2 score, and presenting stroke severity (Fig. 1). No subgroup was identified that either differentially benefitted from statins in preventing recurrent stroke or differentially failed to benefit from statins in preventing in-hospital death.

## 4 Discussion

In this analysis of a nationwide cohort study of stroke patients with atrial fibrillation, we found that statin therapy within 3 months of index stroke failed to reduce recurrent stroke risk in the average 3-year follow-up period. However, statin therapy was associated with reduced risk of future in-hospital death, especially of noncardiovascular death.

Prior randomized trials and observational studies that have shown a benefit of statin therapy in reducing recurrent stroke have focused upon patients whose index ischemic stroke was due to large or small artery atherosclerosis. In SPARCL, statin therapy was associated with a reduction in recurrent stroke risk among patients with index ischemic stroke presumed to have occurred “owing to atherosclerotic causes.”^[3]^ Meta-analyses of statin therapy have also confirmed a beneficial effect of statin therapy in atherosclerotic ischemic stroke patients with regard to stroke recurrence.^[1,9]^ However, these studies generally excluded patient with atrial fibrillation. In this study, we focused on ischemic stroke patient with atrial fibrillation and found no significant difference between the early statin and nonstatin groups in stroke recurrence.

In patients with previous stroke or transient ischemic attack, 2.4% annual recurrent stroke or systemic embolism occurred in the apixaban group, whereas 9.2% annual recurrent stroke or systemic embolism occurred in the aspirin group in a randomized controlled trial.^[10]^ About 40% patients in this study received anticoagulant therapy, mostly warfarin, at baseline and the annual recurrent stroke risk was 8.0%. It was not unreasonable that the annual recurrent stroke risk in this study was higher than patients receiving apixaben in a randomized controlled trial.

Our findings of an association of statin use with reduced mortality are consonant with some prior studies. Several observational studies found that statin treatment is associated with reduced overall mortality in patients with ischemic stroke.^[9,11]^ Also, a cohort study indicated that statins were associated with lower mortality among patients with atrial fibrillation with or without stroke who were less than 80 years old.^[12]^ Recently, the results of 2 hospital-based studies showed that statin treatment was independently associated with reduced mortality specifically in patients with atrial fibrillation-related stroke.^[13,14]^

The mechanisms by which statins may reduce all-cause mortality have not been investigated in detail. Prior studies reporting reduced mortality in atrial fibrillation patients with initial ischemic stroke did not explore whether the effect was mediated by reductions in cardiovascular death, noncardiovascular death, or both.^[13,14]^ Choi and colleagues^[13]^ reported that statins did not reduce the rate of recurrent stroke among atrial fibrillation patients, suggesting that the reduced mortality was not due to reduction in fatal recurrent strokes. We found that lower in-hospital death in the statin therapy group was largely driven by lower noncardiovascular death (e.g., respiratory complication). This may be due to pleotropic effects of statin, such as reducing inflammation.^[1]^ One prior study has found a reduced risk of pneumonia in thrombolyzed stroke patients when statins are administered.^[15]^

Studies have suggested a link between statin use and diabetes in the general population.^[16,17]^ Also, a prior study suggested that statin therapy may increase hemorrhagic stroke risk among ischemic stroke patients.^[3]^ In this study, however, statin therapy was not associated with an increased incidence of future diabetes or hemorrhagic stroke for ischemic stroke patients with atrial fibrillation. One caveat is that the exposure time and cumulative dosage of statin in this study were lower than that in the above clinical trials.

A limitation of the current study is that the reasons for use or nonuse of statins early after the ischemic stroke were not directly available from the analyzed administrative data. Based on a reimbursement regulation in Taiwan, patients were covered to receive statins if they had documented hypercholesterolemia. However, the Taiwan National Health Insurance Administration also suggested that physicians stop or reduce statin dosage when the nation goal cholesterol was reached (total cholesterol<160 mg/dL or low-density lipoprotein cholesterol <100 mg/dL). Patients in the statin group had higher baseline cholesterol levels and only 52% continued statin therapy for more than 1 year. The cholesterol levels of patients with statin discontinuation may return to higher levels, thus increasing the risk of recurrent stroke and showing no benefit of statin therapy in stroke prevention.

This study has additional limitations inherent in studies of administrative datasets. Several established stroke risk factors, such as smoking, lifestyle, and laboratory results, such as cholesterol level, were not available in the claims database. The diagnosis of acute ischemic stroke using the administrative data has high, but not perfect, reliability. In one study of the NHIRD administrative database against the gold standard of the Taiwan Stroke Registry found a positive predictive value of 88.4% and sensitivity of 97.3%.^[18]^ Another study, comparing the NHIRD to detailed diagnoses from review of original hospital medical records, found that 94.5% of acute ischemic stroke patients were assigned “ischemic stroke” as the principal diagnosis in the NHIRD.^[19]^ We could only use in-hospital mortality in the analysis because encrypted NHIRD were not linked to the national death registry. Therefore, out-of-hospital mortality was not captured. Furthermore, the accuracy of the recorded primary cause of in-hospital death in NHIRD is not well validated; thus, this result should be cautiously interpreted. The effect of different statins in preventing recurrent stroke was not analyzed.

In conclusion, statin therapy initiated during the acute to subacute stage of ischemic stroke was not associated with a reduced risk of stroke recurrence in stroke patients with atrial fibrillation. However, statin use was associated with a decreased risk of in-hospital death during follow-up period. Randomized control trials are warranted to provide robust evidence on this issue.
